# Author Correction: HIV-1 Env trimers asymmetrically engage CD4 receptors in membranes

**DOI:** 10.1038/s41586-025-08802-9

**Published:** 2025-02-24

**Authors:** Wenwei Li, Zhuan Qin, Elizabeth Nand, Michael W. Grunst, Jonathan R. Grover, Julian W. Bess, Jeffrey D. Lifson, Michael B. Zwick, Hemant D. Tagare, Pradeep D. Uchil, Walther Mothes

**Affiliations:** 1 Department of Microbial Pathogenesis, Yale University School of Medicine, New Haven, CT USA; 2 AIDS and Cancer Virus Program, Frederick National Laboratory for Cancer Research, Frederick, MD USA; 3 Department of Immunology and Microbiology, The Scripps Research Institute, La Jolla, CA USA; 4 Department of Radiology and Biomedical Imaging, Yale University, New Haven, CT USA

**Keywords:** Virology, Cryoelectron tomography

Correction to: *Nature* 10.1038/s41586-023-06762-6 Published online 22 November 2023

In the version of the article initially published, NIH grant no. U54 AI170856 was missing from the Acknowledgements section and has now been added to the HTML and PDF versions of the article.

