# Supplementary material for: HIV-1 Env trimers asymmetrically engage CD4 receptors in membranes
Source: Nature. 2023 Nov 22;623(7989):1026–33. doi: 10.1038/s41586-023-06762-6 (PMC10686830; doi:10.1038/s41586-023-06762-6)
Supplement: Supplementary file 1 — Supplementary Table 1 (summary table of Cryo-EM data collection) and source data for Extended Data Fig. 1 (uncropped gels). [file 41586_2023_6762_MOESM1_ESM.pdf]

nature portfolio

https://doi.org/10.1038/s41586-023-06762-6

Supplementary information

HIV-1 Env trimers asymmetrically engage CD4 receptors in membranes

In the format provided by the authors and unedited

Supplementary Table 1 | Summary table of Cryo-EM data collection

	Unliganded HIV-1 Env (EMDB-41045)	HIV-1 Env bound to one CD4 (EMDB-29292)	HIV-1 Env bound to two CD4 (EMDB-29293)	HIV-1 Env bound to three CD4 (EMDB-29294)	Consensus structure of HIV-1 Env bound to CD4 (EMDB-29295)
**Data collection and processing**					
Magnification	300	300	300	300	300
Voltage (kV)					
Electron exposure (e–/Å^2)	123	123	123	123	123
Defocus range (μm)	-0.5 (VPP)	-0.5 (VPP)	-0.5 (VPP)	-0.5 (VPP)	-0.5 (VPP)
Pixel size (Å)	2.7	2.7	2.7	2.7	2.7
Symmetry imposed	C3	C1	C1	C1	C1
Initial particle images (no.)	6444	5712	5712	5712	5712
Final particle images (no.)	6339	2341	1418	1953	5712
Map resolution (Å)	9	17	19	16	15
FSC threshold	0.5 cutoff	0.5 cutoff	0.5 cutoff	0.5 cutoff	0.5 cutoff

Source Data of Extended Data Figure 1

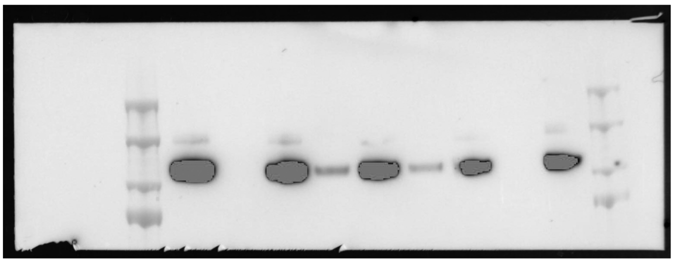

2G12 (anti-gp120)

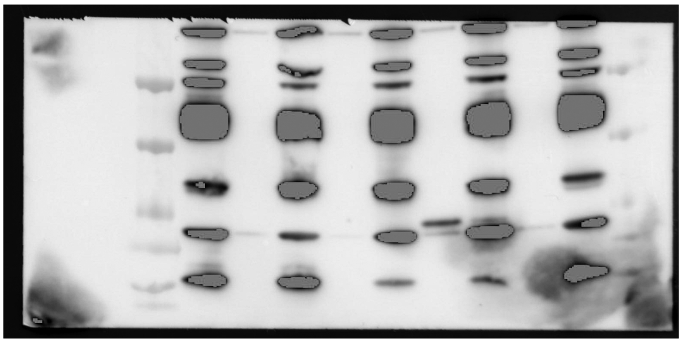

Human anti-HIV-1 Neutralizing Serum
