# Supplementary material for: HIV-1 Env trimers asymmetrically engage CD4 receptors in membranes
Source: Nature. 2023 Nov 22;623(7989):1026–33. doi: 10.1038/s41586-023-06762-6 (PMC10686830; doi:10.1038/s41586-023-06762-6)
Supplement: Supplementary file 2 — Reporting Summary [file 41586_2023_6762_MOESM2_ESM.pdf]

nature portfolio

Corresponding author(s): Walther Mothes, Wenwei Li

Last updated by author(s): Sep 8, 2023

Reporting Summary

Nature Portfolio wishes to improve the reproducibility of the work that we publish. This form provides structure for consistency and transparency in reporting. For further information on Nature Portfolio policies, see our Editorial Policies and the Editorial Policy Checklist.

Statistics

For all statistical analyses, confirm that the following items are present in the figure legend, table legend, main text, or Methods section.

n/a	Confirmed	
☐	☒	The exact sample size (*n*) for each experimental group/condition, given as a discrete number and unit of measurement
☐	☒	A statement on whether measurements were taken from distinct samples or whether the same sample was measured repeatedly
☐	☒	The statistical test(s) used AND whether they are one- or two-sided<br>*Only common tests should be described solely by name; describe more complex techniques in the Methods section.*
☒	☐	A description of all covariates tested
☒	☐	A description of any assumptions or corrections, such as tests of normality and adjustment for multiple comparisons
☐	☒	A full description of the statistical parameters including central tendency (e.g. means) or other basic estimates (e.g. regression coefficient) AND variation (e.g. standard deviation) or associated estimates of uncertainty (e.g. confidence intervals)
☐	☒	For null hypothesis testing, the test statistic (e.g. *F*, *t*, *r*) with confidence intervals, effect sizes, degrees of freedom and *P* value noted<br>*Give P values as exact values whenever suitable.*
☒	☐	For Bayesian analysis, information on the choice of priors and Markov chain Monte Carlo settings
☒	☐	For hierarchical and complex designs, identification of the appropriate level for tests and full reporting of outcomes
☒	☐	Estimates of effect sizes (e.g. Cohen's *d*, Pearson's *r*), indicating how they were calculated

Our web collection on statistics for biologists contains articles on many of the points above.

Software and code

Policy information about availability of computer code

Data collection	Cryo-ET data were collected on Titan Krios (Thermo Fisher) by an automated EM data acquisition software SerialEM 3.8.
Data analysis	Tomograms were motion-corrected using Motioncorr2, aligned and reconstituted by IMOD 4.11. Subtomogram averaging analyses was performed with I3 0.9. The structures were visualized and segmented in the UCSF ChimeraX 1.3. Local resolutions of the structures were measured by Resmap 1.1.4. Molecular Dynamics Flexible Fittings (MDFF) were performed in NAMD 3.0 Alpha with CHARMM36 force-field parameters. Custom scripts for multiple distance spatial cluster analysis, kernel density heatmaps and histogram profiles were generated with Python 3.8.

For manuscripts utilizing custom algorithms or software that are central to the research but not yet described in published literature, software must be made available to editors and reviewers. We strongly encourage code deposition in a community repository (e.g. GitHub). See the Nature Portfolio guidelines for submitting code & software for further information.

Data

Policy information about availability of data

All manuscripts must include a data availability statement. This statement should provide the following information, where applicable:

- Accession codes, unique identifiers, or web links for publicly available datasets
- A description of any restrictions on data availability
- For clinical datasets or third party data, please ensure that the statement adheres to our policy

TThe cryo-EM structures have been deposited to the Electron Microscopy Data Bank (EMDB). Cryo-ET structural maps for HIV-1 Env bound to one, two and three CD4 molecules, the consensus structure of HIV-1 Env bound to CD4 receptors in membranes and the unliganded Env trimer have been deposited with accession codes EMD-29292, EMD-29293 and EMD-29294, EMD-29295 and EMD-41045, respectively.

Human research participants

Policy information about studies involving human research participants and Sex and Gender in Research.

Reporting on sex and gender	n/a
Population characteristics	n/a
Recruitment	n/a
Ethics oversight	n/a

Note that full information on the approval of the study protocol must also be provided in the manuscript.

Field-specific reporting

Please select the one below that is the best fit for your research. If you are not sure, read the appropriate sections before making your selection.

☒ Life sciences ☐ Behavioural & social sciences ☐ Ecological, evolutionary & environmental sciences

For a reference copy of the document with all sections, see nature.com/documents/nr-reporting-summary-flat.pdf

Life sciences study design

All studies must disclose on these points even when the disclosure is negative.

Sample size	168 tomograms containing 857 membrane-membrane interfaces were captured in available electron microscopy time. Subtomogram averaging structures from 5712 particles reached the resolution beyond 20 Å to support the conclusions in the manuscript.
Data exclusions	No data were excluded from analyses.
Replication	Different grids from distinct sample preparations were imaged.
Randomization	Membrane-membrane interfaces between HIV-1 and MLV particles were randomly formed and picked with no bias. Structure resolutions were determined by two random halves of the data set.
Blinding	The investigators were not blinded during data collection or during analysis. Groups are not relevant to this work.

Reporting for specific materials, systems and methods

We require information from authors about some types of materials, experimental systems and methods used in many studies. Here, indicate whether each material, system or method listed is relevant to your study. If you are not sure if a list item applies to your research, read the appropriate section before selecting a response.

Materials & experimental systems

n/a	Involved in the study
☐	☒ Antibodies
☐	☒ Eukaryotic cell lines
☒	☐ Palaeontology and archaeology
☒	☐ Animals and other organisms
☒	☐ Clinical data
☒	☐ Dual use research of concern

Methods

n/a	Involved in the study
☒	☐ ChIP-seq
☒	☐ Flow cytometry
☒	☐ MRI-based neuroimaging

Antibodies

Antibodies used

2G12 (ARP-1476, Lot 140426), Human anti-HIV-1 Neutralizing Serum (ARP-1983, Lot 4/2/91), HRP Goat Anti-Human Ig (Cat. No. 2010-05, Lot D7511-M985C, SouthernBiotech)

Validation

Human monoclonal antibody 2G12
Antibody class: IgG1κ
ARP-1476 is a recombinant monoclonal antibody to HIV-1 gp120.
This antibody was produced in a recombinant Chinese Hamster Ovary (CHO) cell expression system and purified by Protein A affinity chromatography. This antibody originates from an HIV-1 positive human donor. This antibody neutralizes a broad variety of SHIV variants, HIV-1 laboratory strains and primary isolates. The epitope is conformational and carbohydrate dependent. It is directed against N-linked glycans in the C2, C3, V4, and C4 domains of gp120.

Human anti-HIV-1 Neutralizing Serum
Host: Human
ARP-1983 was pooled from four separate bleeds from the same human immunodeficiency virus type 1 (HIV-1)-positive patient. The antiserum was diluted with PBS and distilled water prior to lyophilization. ARP-1983 is at a 1:2 final dilution of the original sample. ARP-1983 is provided as 1 ampule of sterile, heat inactivated, lyophilized antiserum.

HRP Goat Anti-Human Ig
Isotype: Goat IgG
Isotype Control: 0109-05
Specificity: Reacts with the heavy and light chains of human IgG, IgM, and IgA
Source: Pooled antisera from goats hyperimmunized with human IgG, IgM, and IgA
Cross Adsorption: None; may react with immunoglobulins from other species
Purification Method: Affinity chromatography on pooled human immunoglobulins covalently linked to agarose
Conjugate: HRP (Horseradish Peroxidase)

Eukaryotic cell lines

Policy information about cell lines and Sex and Gender in Research

Cell line source(s)

HEK293T cells (ATCC)
TZM-bl (from NIBSC, Centre For AIDS Reagents, ARP5011)
HIV-1BaL/SUPT1-CCR5 cells (cell line ID #CLN204, generated by Jeffrey Lifson laboratory, AIDS and Cancer Virus Program, Frederick National Laboratory for Cancer Research, Frederick, MD, USA)
ADA.CM.755*/HEK293T (generated by Michael Zwick laboratory, Department of Immunology and Microbiology, The Scripps Research Institute, La Jolla, CA, USA)

Authentication

Virus HIV-1BaL were generated from HIV-1BaL/SUPT1-CCR5 cells. Virus HIV-1ADA.CM.755* were generated from ADA.CM.755*/HEK293T by transfecting HIV-1 gagpol plasmid. MLV-CD4 viral particles were generated from HEK293T cells by co-transfecting MLV gagpol and CD4 receptor expressing plasmid. Plasma membrane blebs were produced from TZM-bl cell line incubated with blebbing buffer.

Mycoplasma contamination

The cell lines were not contaminated by mycoplasma.

Commonly misidentified lines
(See ICLAC register)

None
